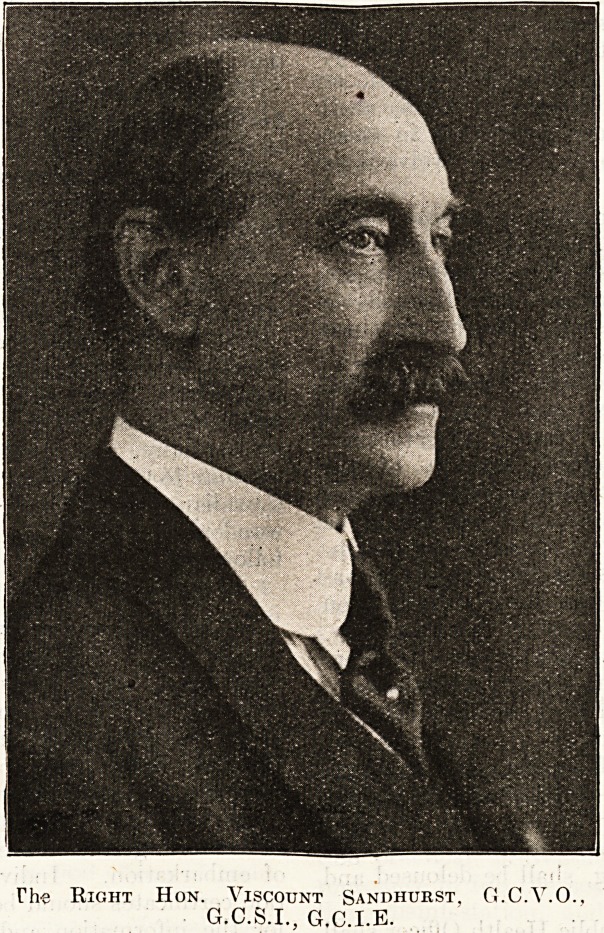# The Late Viscount Sandhurst

**Published:** 1921-11

**Authors:** 


### THE LATE VISCOUNT SANDHURST.

### THIRTY YEARS' WORK FOR HOSPITALS.

Viscount Sandhurst, whose death occurred on November 2, after an illness which had lasted some time, is probably best known to the hospital world as Treasurer of St. Bartholomew's Hospital, an office which he had filled since November 5, 1908, though for the first two years his other public duties necessitated the delegation of the Treasurer's work.

Lord Sandhurst first became prominent in the hospital world as Chairman of the Lords' Committee on the Metropolitan Hospitals, which, as the result of his inspiration, was appointed in 1890 to inquire into the constitution and working of the medical charities of the Metropolis. The volumes containing the evidence given before this Committee, and its report which was issued in June 1892, and may be termed a hospital classic, must be well known to hospital administrators and the medical profession, as are the activity and interest displayed by Lord Sandhurst throughout the Committee's lengthy sittings.

The Right Hon. Viscount Sandhurst, G.C.V.O., G.C.S.I., G.C.I.E.

After his chairmanship of the Lords' Committee, Lord Sandhurst became Chairman of the Middlesex Hospital, an office he held for eight and a-half years in all, though its continuity was interrupted by his appointment as Governor of Bombay, where he remained from 1895 to 1900. During this period it was his lot to deal with two famines and four epidemics of plague, and on each of these occasions he threw himself into the work of relief with conspicuous courage and devotion. These qualities, with those of sympathy, thoroughness, and extraordinary consideration for those with whom he worked, were characteristic of the man throughout the whole of his life.

On returning to England, Lord Sandhurst resumed the chairmanship of the Middlesex Hospital. His direct association with St. Bartholomew's Hospital began in 1903, when he served on the Lord Mayor's Committee to consider the question of its site and rebuilding. The Committee reported in favour of the retention of the present site and of the scheme of rebuilding suggested by the Governors; the reconstruction of St. Bartholomew's was in consequence proceeded with, and necessary extensions provided for by the purchase of the Christ's Hospital site. It was typical of Lord Sandhurst that when he became Treasurer in 1908 one of his first acts was to appoint a Committee to inquire into the whole working of the Hospital.

Activity, both financial and administrative, has characterised the whole period of his treasurership. The Peace Year Commemoration Appeal is the most recent of the great efforts made under his auspices, but despite its undoubted success, he confessed in 1919 to grave doubts "as to whether the funds necessary to meet the enormously increased annual needs, apart from imperative capital expenditure, will be forthcoming from voluntary sources, and other means of supplementing the Hospital's income may have to be sought." Apart from the revenue derived from its invested property, St. Bartholomew's has to raise a voluntary income of £70,000 a year, and in 1920 the Governors obtained authority from the Charity Commissioners to establish the principle of patients' payments.

Lord Sandhurst had been in failing health for some time, and at the Court of Governors in July last, on his reappointment as Treasurer, he made it clear that he would not be able to continue in office after the end of the present year. His has been a strenuous life of service to the Crown and to his country, and his death at the early age of sixty-six will be widely deplored. In addition to his other hospital work he was President of the British Hospitals Association from 1916 to 1920, and in the House of Lords he rendered considerable service to nurses in connection with State Registration.

## Figures and Tables

**Figure f1:**